# Role of capsid proteins in parvoviruses infection

**DOI:** 10.1186/s12985-015-0344-y

**Published:** 2015-08-04

**Authors:** Mengyu Tu, Fei Liu, Shun Chen, Mingshu Wang, Anchun Cheng

**Affiliations:** Institute of Preventive Veterinary Medicine, Sichuan Agricultural University, Wenjiang District, Chengdu City, Sichuan Province 611130 China; Avian Disease Research Center, College of Veterinary Medicine of Sichuan Agricultural University, Wenjiang District, Chengdu City, Sichuan Province 611130 China; Key Laboratory of Animal Disease and Human Health of Sichuan Province, Sichuan Agricultural University, Wenjiang District, Chengdu City, Sichuan Province 611130 China

**Keywords:** Parvovirus, capsid proteins, functions, viral infection

## Abstract

The parvoviruses are widely spread in many species and are among the smallest DNA animal viruses. The parvovirus is composed of a single strand molecule of DNA wrapped into an icosahedral capsid. In a viral infection, the massy capsid participates in the entire viral infection process, which is summarized in this review. The capsid protein VP1 is primarily responsible for the infectivity of the virus, and the nuclear localization signal (NLS) of the VP1 serves as a guide to assist the viral genome in locating the nucleus. The dominant protein VP2 provides an “anti-receptor”, which interacts with the cellular receptor and leads to the further internalization of virus, and, the N-terminal of VP2 also cooperates with the VP1 to prompt the process of nucleus translocation. Additionally, a cleavage protein VP3 is a part of the capsid, which exists only in several members of the parvovirus family; however, the function of this cleavage protein remains to be fully determined. Parvoviruses can suffer from the extreme environmental conditions such as low pH, or even escape from the recognition of pattern recognition receptors (PRRs), due to the protection of the stable capsid, which is thought to be an immune escape mechanism. The applications of the capsid proteins to the screening and the treatment of diseases are also discussed. The processes of viral infection should be noted, because understanding the virus-host interactions will contribute to the development of therapeutic vaccines.

## Background

Parvoviruses infect a wide range of species, including birds and mammals. The virus replicates in the nucleus, although some of the parvoviruses require a helper virus to replicate [1, 2]. The viral replication must occur in mitotic cells because the virus must use a polymerase to effectively proliferate. Based on the host specificity, the *Parvoviridae* family is divided into two groups, the *Parvovirinae* and the *Densovirinae. Parvovirinae* infects vertebrates, whereas *Densovirinae* infects only invertebrates. Recently, a new classification of the *Parvoviridae* family was proposed, and the latest subfamily designation, the *Parvovirinae*, now has eight genera: *Amdoparvovirus*, *Aveparvovirus*, *Bocaparvovirus*, *Copiparvovirus*, *Dependoparvovirus*, *Erythroparvovirus*, *Protoparvovirus* and *Tetraparvovirus* [3]. The human parvovirus B19 (B19) causes a serious autoimmune disease in children [4]. An infection with the virus during pregnancy can lead to hydrops foetalis and foetal loss or congenital infection [5]. The adeno-associated viruses (AAVs) are nonpathogenic and replication-defective viruses in the *Parvoviridae* family, with twelve distinct AAV serotypes and more than 100 recombinant species [6]. Additionally, the AAVs cannot effectively complete replication without the help of viruses such as adenoviruses or herpesviruses [2]. In animals, the most common symptoms are gastroenteritis and diarrhoea [7–9]. In general, parvoviruses agglutinate erythrocytes, but the goose parvovirus (GPV) is an exception; the GPV cannot agglutinate red blood cells but agglutinates cattle sperm [8].

Because of the specific immunogenicity, capsid proteins have great potential for the development of vaccines. The baculovirus expression system has been widely used to produce virus-like particles (VLPs), which share a immunogenicity that is similar to native viruses and that can be applied to further study of the functional aspects [10, 11]. Generally, the VLPs are well immunogens which can induce a strong and specific antiviral immune response. Although the analyses of the structural and functional aspects of the VLPs have always attracted much attention, there is no systematic description of the function of each capsid protein during the viral invasion.

In this paper, the genome and the encoding proteins of parvoviruses and the roles of capsid proteins of the viruses in viral infection are summarized, leading to suggestions for possible mechanisms to explain the interactions between virus and host. Moreover, we reviewed the application of recombinant viral capsids to the treatment of diseases.

### The genome and encoding proteins of parvoviruses

The parvovirus genomes are approximately 5.0 kb in length and is enclosed within an icosahedron capsid (T = 1), which is 18–26 nm in diameter. At both ends of the genome, inverted terminal repeats (ITR) are formed by palindromic sequences, which are assembled into different shapes of a hairpin structure (depending on virus). In most of the parvoviruses, the viral DNA encodes two open reading frames (ORF). The ORF1 encodes nonstructural proteins (NS), and the ORF2 encodes two or three viral particle (VP) proteins which assemble the viral capsid; the VP proteins share a common termination codon. However, a few parvoviruses possess more than two ORFs, including, for example, members of the *Bocaparvovirus*, which have an extra ORF that encodes a nuclear phosphoprotein NP1 [12–15]. The NS protein is a replicate protein that control genomic replication, is cytotoxic to host cells and is a cause of apoptosis [16, 17]. Successful examples of continuously producing viral particles in cell lines did not occur until a lac repressor-operator system was included, which first successfully overcame the cytotoxicity problem and allowed for stringent regulation of these proteins [18]. The NS1 protein has several replication-related regions, for example, a DNA-binding region, an ATP binding region, a helicase domain and a transactivation domain [16]. In the B19, AAV, minute virus of mice (MVM), canine parvovirus (CPV), porcine parvovirus (PPV), bovine parvovirus (BPV) and GPV, the VP1 contains the entire sequence of the VP2. Compared with the VP2, the VP1 has an extra length of ~140 amino acids at the N-terminal, with a phospholipase A_2_ (PLA_2_) domain and an NLS. The VP2 constitutes the primary component of the capsid protein and is highly conserved. The VP3 is a cleavage product that appears after the translation of VP2, and not all the parvoviruses have this protein. Although sequence diversity exists among the different species, these viruses have similar structures as determined by the three-dimensional structures, such as for B19 [19], AAV [20–27], MVM [28], CPV [29, 30], BPV [9] and the Aleutian mink disease virus (ADV) [31].

### Role of parvovirus capsid in viral infection

**Fig. 1:**
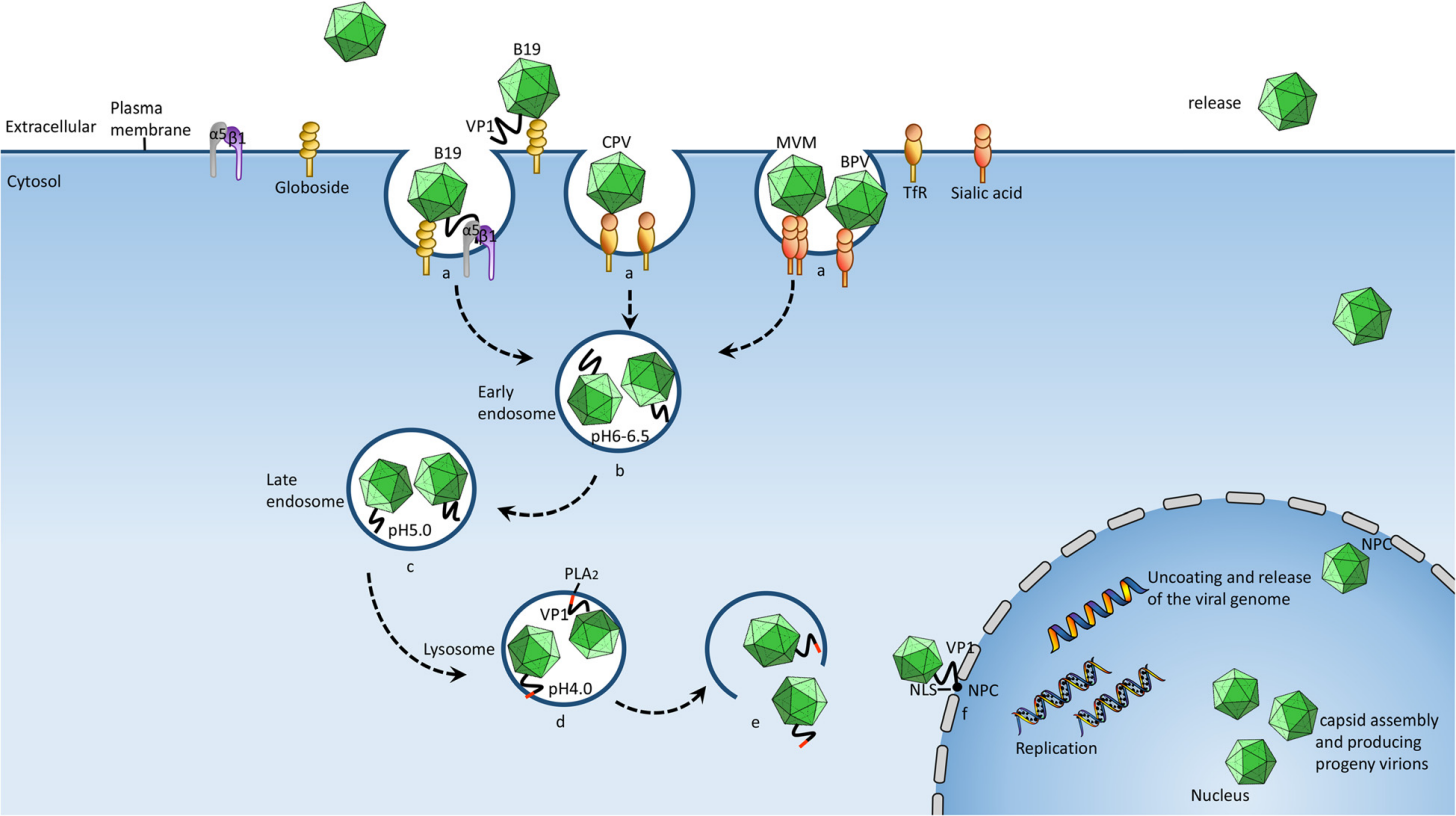
A schematic of the parvovirus infection process, mediated by the Clathrin-dependent endocytic pathway. The internalization of the virus is mediated by the endocytosis pathway, primarily through the following steps. **a** Receptor binding-mediated internalization. The cellular receptor for parvovirus B19 is globoside, the cellular receptor for CPV is transferrin receptor (TfR), and the cellular receptor for MVM and BPV is sialic acid. **b** Form of the early endosome (pH 6.0-6.5). **c** Transformed into the late endosome at lower pH conditions (pH 5). **d** Transformed into the lysosome and the activation of phospholipase A_2_ (PLA2) (pH 4.0). **e** The activated PLA2 destroys the integrity of the lysosomal membrane. Thus, the viruses are released into the cytosol. **f** The viruses are transported towards the nucleus and target on the karyotheca with the help of the VP1 nuclear localization signal (NLS). The movement of microtubule and actin filaments are involved in the entire process of viral infection, from the early endosome to the peripheral nucleus. The viral genome begins to replicate and assemble after trafficking through the nuclear pore complex (NPC). The matured virions finally move through the NPC and are released extracellular

The multifunctional capsid is responsible for the adsorption on and the entrance into the host cell, intracellular transport and localization, viral egress and induction of the immune response. Figure 1 shows the process of viral infection. In the following sections, the function of each capsid protein during the viral infection will be discussed.

### VP1 is responsible for viral infectivity

In most parvoviruses, the VP1 N-terminal changes location from internal to being external of the capsid when exposed to some extreme chemical and physical conditions, such as heat or low pH. Moreover, the heat-induced exposure of the VP1 N-terminal is an irreversible process [32, 33]. The conformational changes of the capsid VP1 and the exposure of the VP1 N-terminal play a considerable role in viral infection. In previous studies with B19, a recombinant capsid VP1 N-terminal with a unique region (VP1u) was readily recognized by a specific monoclonal antibody, whereas the natural capsid was not recognized. Furthermore, after a heat or a low-pH treatment, the native VP1u was accessible to the antibody [34]. These results revealed a primarily conformational difference between the native and recombinant virion for VP1u and preliminarily showed that the B19 VP1u peptides were originally located inside the capsid. Additionally, the CPV and MVM virions exposed the VP1 N-terminal during endosomal trafficking, which also argued that the VP1 N-terminal was initially hidden in the viral capsid [33, 35]. Mutational experiments confirmed that the VP1 N-terminal of the MVM directly affected the infectivity of the virus [36]. Additionally, the direct effect the VP1 N-terminal on infectivity was observed with AAV-2. The microinjection of wild-type AAV-2 directly into the cytoplasm resulted in low infectivity, but the infectivity improved with the exposure of the VP1 N-terminal, which strongly suggested again that the VP1 N-termini are essential for the intracellular virion to initiate infection [37]. Additional studies revealed that B19 VP1u became external after primary cellular receptor attachment [38, 39]. Furthermore, the VP1u mediated internalization was a highly restricted process and only occurred in the permissive erythroid lineages, which suggested a viral tropism and pathogenesis [40].

The NLS is a nuclear localization signal that assists in the navigation of viruses to the nucleus, which leads to further nuclear translocation. A previous study identified a typical NLS on the VP1 N-terminal residues 4–13 in the CPV and demonstrated that the nuclear translocation was an ATP-dependent process [41]. An NLS was also identified in the MVM [36]. Moreover, the VP1 N-terminal region of the MVM is composed of four basic amino acid clusters, BC1, BC2, BC3 and BC4. Mutational and biochemical studies determined that BC1 and BC2 exhibited a great potential for nuclear transport [36]. The sequences of the BCs are highly conserved among parvoviruses; thus, we inferred that the BC elements, which contain the NLS, most likely participate in the nuclear transport in other representative parvoviruses, as well. A recent study showed that the PPV was distinctly different from the closely related parvoviruses because the PPV had more than one activated NLS, including a novel nuclear localization motif (NLM) [42]. One of the NLSs is typically located on the VP1 N-terminal and plays a role in the early steps of infection, whereas the other NLS is a novel NLM that targets the VP2 trimers to the nucleus late in the infection [42]. Additionally, in the genus *Bocaparvovirus*, the NLSs display some distinct properties in comparison with the other parvoviruses. Direct experiments suggest that the human bocavirus NP1 protein possesses a nonconventional NLS and is capable of transporting β-galactosidase fusion proteins to the nucleus [43], which indicates that the NP1 plays a role in nuclear translocation. Notably, in ADV, the VP1 binds the DNA, and the virus can not replicate efficiently in the nucleus because of the absence of the NLS [44].

In the majority of parvoviruses, the VP1 N-termini have a secretory PLA_2_ (sPLA_2_s) homology domain, which contains the catalytic site of a sPLA_2_ and a conserved Ca^2+^ binding loop. However, a few of the viruses do not have this motif. The PLA_2_ is a lipolytic enzyme that destroys the membrane and allows the viruses to escape from the lysosome. Additionally, the PLA_2_ is located initially within the native capsid but is then turned to the outside after heat or pH treatments [34]. In addition to the conserved amino acids within the VP1u PLA_2_ motif, some nonconserved amino acid residues around the VP1u also have an effect on PLA_2_ activity. The B19 mutants of the PLA_2_ motif showed a significant decrease in PLA_2_ activity and viral infectivity, which suggested that the PLA_2_ played an important role in the B19 life cycle [45]. Furthermore, in their study, Deng *et al*. demonstrated that the integrity of the cell membrane was destroyed by PLA_2_ in a B19 infection. When the UT7-Epo cells were incubated with purified VP1u proteins, the cell morphology changed with time until finally the change was abrupt, resulting even in death, with an increase in the VP1u treatment. However, in comparison, the cells with mutant VP1u proteins or those in the control treatment did not change [46]. The identical effect was also validated for the AAV [47]. Additionally, the structural and conformational changes that occurred during the VP1 N-terminal externalization from the inner capsid to the outside were physically revealed [48]. In a comparison with humans, the question remains whether the same process occurs in animals. In the MVM, the phosphorylation of PLA_2_ activity also influenced the stage of virus released from the endosome. After A9 cells were transfected with VP1u mutant infectious clones, the PLA_2_ activity and the MVM infectivity were both abrogated [49]. Similarly in CPVs, when the capsid was incubated with PLA_2_ inhibitors, the infectivity of the CPVs was significantly reduced and the endosome membrane permeability was changed during the period of CPV infection [35]. These results also demonstrated that PLA_2_ activity is essential for effective infection.

### VP2 participates in the receptor recognition and in nuclear translocation

Viral infection begins with the adhesion of the virus to a cell surface receptor [50]. In viruses, there is also an “anti-receptor” that is found in the parvovirus VP2 protein, which attaches to the cell receptor and begins internalization (Table 1). With the use of the cryo-electron microscopy technique at the resolution of 8-Å, a depression at the icosahedral threefold axes of the B19 VP2 capsid was found, which bound to the cellular receptor globoside [51]. In the AAV-2, the heparin sulphate proteoglycan is a dominant receptor [52], which shares a common receptor α5β1 with B19. When the AAV-2 was combined with heparin, at ~18 Å resolution under cryo-electron microscopy, the conformational changes of the capsid were revealed; the tip of the protrusions on the three-fold axes became flat, and the top of the channel located on top of the five-fold axes grew broader [53]. In contrast to B19, there is a distinct “spike” on the three-fold axes of CPV VP2, based on observations of the 3D structure. On the VP2 capsid surface, there is a putative receptor-binding region located in a depression with a canyon-like shape. Moreover, one of the binding regions is located between the two icosahedral three-fold axes, and the other binding region is on the five-fold axes [30]. The BPV capsid VP2 showed a common parvovirus feature, obvious protrusions encircling the threefold axes, which indicated a potential site for receptor recognition [9]. For ADV, the 3D structure of VP2 capsid have been determined to 22 Å resolution, a dimple at the twofold axes was indicated to be involed in the recognition of cellular receptor [31].

**Table 1:** The subfamily *Parvovirinae*: the interaction between the virus and the host

Genus	Virus	Host	Cellular Receptor	Invasion pathway	Binding site	Accession number
*Amdoparvovirus*	*Aleutian mink disease virus*	Mink	Sialic acid	-	A protrusions on threefold axes and the wall of the dimples on twofold axe	M20036
*Bocaparvovirus*	*Bovine parvovirus*	Bovine	α2-3 O-linked sialic acid	Clathrin-dependent endocytic pathway	-	M14363
*Dependoparvovirus*	*Adeno-associated virus*	Human	Heparan sufate proteoglycan, sialic acid, aVβ5 integrin	Clathrin-dependent or independent internalization	-	
	*Goose parvovirus*	Goose, Muscovy duck	-	-	-	U25749
*Erythroparvovirus*	*Human parvovirus B19*	Human	P antigen, α5β1, ku80	Clathrin-dependent endocytic pathway	At depression of the three fold axis	NC_000883
*Protoparvovirus*	*Canine parvovirus*	Dogs, cats	Transferrin receptor	Clathrin-dependent endocytic pathway	A distinct “spike” on threefold axes	EF011664
	*Minute virus of mice*	Rodents	α2-3 and α2-8 N-linked Sialic acid	Both clathrin- and lipid-raft mediated endocytosis	At the depression of twofold axes and the floor of twofold axes depression	V01115
	*Porcine parvovirus*	Swine	α2-3 N- and O-linked Sialic Acid	Macropinocytosis and Clathrin-dependent endocytic pathway	-	M38367
	*Mink enteritis virus*	Mink	Transferrin receptor	-	-	D00765

Notably, a new NLS of the B19 virus was located on the VP2 N-terminal, which could facilitate nuclear transport [54]. In the case of MVM, the VP2 N-terminal also acted as a nuclear export signal (NES) [55]. In further studies, when the temperature reached a specific level, the VP2 N-terminal region of the MVM transferred from the inner capsid through the five-fold axes to the outside of the virion [56]. After a series of conformational changes and the exposure of the NLS and NLM, the VP1 cooperated with the VP2 and formed a trimer with the assistance of VP2-chaperone activity to finally traffic through the nuclear pore complex [57]. Additionally, the VP2 N-terminal was phosphorylated during the late stage of the MVM life cycle, and therefore, the viruses spread to neighbouring cells efficiently prompted by the activated VP2. Miller *et al.* hypothesised that the N-terminal of the VP2 together with the NS2 had an influence on the viral egress from the nucleus [58]. In the case of ADV, a D534 residue of the VP2 enabled the ADV-G to replicate in mink, which induced a continuous immune response [59].

### VP3 function as a capsid scaffold?

The VP3 generally occurs only when the viral genome has completed the capsid assembly and packaging. In the MVM, the VP3 is generated from the cleavage of VP2 at approximately 25 amino acids from the N-terminal. A trypsin digestion experiment demonstrated that this proteolytic reaction occurred only in the mature virion, with an intact genome. Although the VP1 has an identical proteolytic site, cleavage does not occur [60]. An identical proteolytic phenomenon also appeared in the ADV, but with some dissimilarity. Previous studies showed that during an ADV infection or when the viral capsid only was expressed an extra, an unknown 26 kDa protein was produced, which was recently identified as a split product of the capsid VP1 and VP2. Based on the results, the caspase family was activated during the expression of the ADV capsid proteins, and the caspase-7 response was to specifically cleavage the capsid at a distinct site [61].

The studies on VP1 and VP2 are sufficiently comprehensive, but research on VP3 is lacking, and further studies are required to reveal the discrete functions of this protein. For birds, the VP3 is a prominent protein in both GPV and Muscovy duck parvovirus (MDPV), in which the VP3 induced a distinct immune response [62]. Recombinant GPV capsid proteins were expressed and purified to apply to an *in vivo* assay, and all of the VLPs induced a strong immune response in the susceptible geese, whereas the VLPs-VP2 and VLPs-VP3 induced higher concentrations of neutralized antibodies than the VLPs-VP1 [11].

Notably, a 23 kDa protein encoded by the AAV ORF2 promoted the assembly of the VP3 capsid and was named as one of the assembly-activating proteins (AAP). When the AAP was expressed, some of the VP3 was transformed into the nucleus to form the capsid [63], which indicated that the AAP enabled the transport of VP proteins to the nucleoli and was required for the capsid package. The inference was that the VP3 was responsible for the capsid assembly and the virion stability. However, the role of VP3 in the parvovirus life cycle remains to be fully understood.

### Applications of capsid proteins

For the viral capsid, engineers conducted a variety of innovations. Other viruses, for example, a non-enveloped virus in the *Picornavirus* family that causes foot-and-mouth disease (FMD), have much in common with parvoviruses, and interference against the VP1 structural protein successfully produced a protective effect in both cells and suckling mice [64]. This result indicated that RNAi technology might provide a therapeutic measure to treat virus infection. In the parvoviruses, the dominant antigenic determinants are located on the capsid. Langeveld *et al*. used an immunofluorescent assay to analyse the B-cell epitopes on structural proteins and, based on this research, obtained the CPV polypeptide vaccine [65]. Moreover, the VLPs-VP2 of the CPV expressed in *Escherichia coli* and inoculated in mice showed subtle differences between the native viruses and the VLPs in neutralizing antibody titres, in addition to the immune response of the T-cells [66]. The CPV VLPs shared properties closely with the native virus and could be profitable candidates for therapeutic vaccines. Furthermore, researchers used the relation between CPV and its cellular receptor TfR (also exist in human cells) to explore a potential nano-container for tumor targeting. In their study, the accessible lysines on the viral capsid were derivatized with dye molecules. After incubation with TfR expression or those that lacked the TfR expression cell lines, Researchers found that the internalization of the labelled CPV-VLPs was observed only in the TfR expression cell lines [67], which indicated that the CPV-VLPs could act as a target delivery substance.

Adeno-associated viruses (AAVs) are nonpathogenic members of the *Parvoviridae* family. The last 10 years has witnessed a surge of studies on the use of AAVs as vectors. For example, a recombinant triple-tyrosine mutant AAV-2 vector significantly increased the efficiency of gene transduction, approximately three-fold compared with the wild type [68]. Thus, we could minimize the therapeutic dose and produce a preferred level of protection. More recently, Giridhara *et al*. found that AAV vectors activated the NF-κB pathway, which suggested an application in gene therapy [69]. Furthermore, Mirta *et al.* constructed a recombinant AAV capsid vector, which contained a fragment of a tumour-targeting sequence, and the modified mutant resulted in high transduction in tumour cells, but the transduction in 293 T cells was poorly induced. By contrast, the native AAV capsid was highly expressed in the 293 T cell lines, and this altered tropism suggested a strategy to develop an in vivo-targeted vaccine [70].

## Conclusions

Although the size and structure of the parvovirus capsid are simple, each component of the capsid performs a vital function in the life cycle of a virus. The components of the capsid participate in cellular recognition, the endosomal pathway and nuclear trafficking and even induce the immune response. In this review, we summarized the roles of the capsid in viral infection and also the potential applications of recombinant viral capsids in the treatment of disease. In the last several years, studies on the capsids of the parvoviruses have grown in number. Nevertheless, the exact function of the VP3 must be fully elucidated. Recently, many new virus strains were categorized into *Parvoviridae* after the revision of the family; however, the studies on these strains remain at the bioinformatics analysis stage. In the future, more functional-type research must be developed. Furthermore, the study of virus-host interactions remains a major challenge, and an understanding of the mechanisms of the interplay between the virus and the host will contribute to the development of future treatments. Additionally, the safety of the engineered vaccines requires consideration.

## Abbreviations

PRRs: Pattern recognition receptors
VLP: virus-like particle
NS: Non-structural
VP: Viral particle
B19: Human parvovirus B19
AAV: Adeno-associated viruses
GPV: goose parvovirus
ORF: Open reading frames
ITR: Inverted terminal repeats
MVM: Minute virus of mice
CPV: Canine parvovirus
PPV: Porcine parvovirus
BPV: Bovine parvovirus
NLS: Nuclear localization signal
PLA2: Phospholipase A2
ADV: Aleutian mink disease virus
VP1u: VP1 N-terminal unique region
NLM: Nuclear localization motif
NPC: Nuclear pore complex
